# How much does the typical ecological meta‐analysis overestimate the true mean effect size?

**DOI:** 10.1002/ece3.9521

**Published:** 2022-11-15

**Authors:** Jeremy W. Fox

**Affiliations:** ^1^ Department of Biological Sciences University of Calgary Calgary Alberta Canada

**Keywords:** best linear unbiased predictions, effect size, hierarchical model, meta‐analysis, meta‐meta‐analysis, random effects, shrinkage

## Abstract

Many primary research studies in ecology are underpowered, providing very imprecise estimates of effect size. Meta‐analyses partially mitigate this imprecision by combining data from different studies. But meta‐analytic estimates of mean effect size may still remain imprecise, particularly if the meta‐analysis includes a small number of studies. Imprecise, large‐magnitude estimates of mean effect size from small meta‐analyses likely would shrink if additional studies were conducted (regression towards the mean). Here, I propose a way to estimate and correct this regression to the mean, using meta‐meta‐analysis (meta‐analysis of meta‐analyses). Hierarchical random effects meta‐meta‐analysis shrinks estimated mean effect sizes from different meta‐analyses towards the grand mean, bringing those estimated means closer on average to their unknown true values. The intuition is that, if a meta‐analysis reports a mean effect size much larger in magnitude than that reported by other meta‐analyses, that large mean effect size likely is an overestimate. This intuition holds even if different meta‐analyses of different topics have different true mean effect sizes. Drawing on a compilation of data from hundreds of ecological meta‐analyses, I find that the typical (median) ecological meta‐analysis overestimates the absolute magnitude of the true mean effect size by ~10%. Some small ecological meta‐analyses overestimate the magnitude of the true mean effect size by >50%. Meta‐meta‐analysis is a promising tool for improving the accuracy of meta‐analytic estimates of mean effect size, particularly estimates based on just a few studies.

## Graphical abstract

Meta‐analytic estimates of mean effect size can be imprecise and overestimate effect magnitude, particularly if the meta‐analysis includes few studies. Here, I use meta‐meta‐analysis (meta‐analysis of meta‐analyses) to quantify and correct for overestimation of the magnitude of mean effect sizes in ecological meta‐analyses. The typical (median) ecological meta‐analysis overestimates the magnitude of the mean effect size by ~10%, and some meta‐analyses overestimate the magnitude of the mean effect size by >50%.

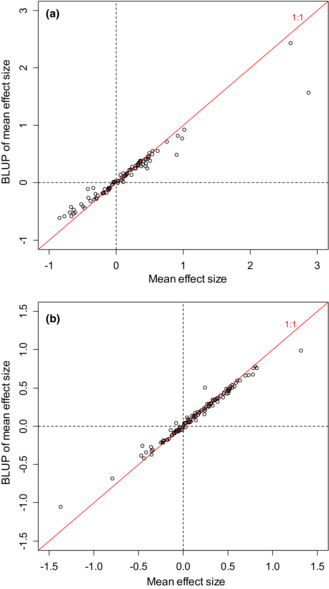

## 1 INTRODUCTION

Primary research studies in ecology are underpowered: most provide very imprecise estimates of effect size and so have a low probability of detecting an effect of typical magnitude (Cleasby et al., 2021; Jennions & Møller, 2003; Lemoine et al., 2016; Parris & McCarthy, 2001; Smith et al., 2011; Yang et al., 2022). Meta‐analysis partially mitigates this imprecision and associated lack of power (Yang et al., 2022). Combining the results of many primary research studies into a meta‐analysis produces a more precise estimate of the mean effect size, increasing power against the null hypothesis of zero mean (Yang et al., 2022).

However, even meta‐analytic estimates of mean effect size remain at least somewhat imprecise, particularly estimates from small meta‐analyses. The median ecological meta‐analysis comprises just 64 effect sizes from just 24 primary research studies (information extracted from data compiled by Costello & Fox, 2022). Just by chance, small meta‐analyses are more likely than large ones to produce imprecise, large‐magnitude estimates of mean effect size. Such imprecise, large‐magnitude estimates are likely to overestimate the true absolute magnitude of the mean effect size. If additional studies were conducted, the estimated mean effect size likely would shrink in magnitude due to regression to the mean (Kelly & Price, 2005). If possible, we would like to quantify and correct this regression to the mean. How much does the typical ecological meta‐analysis overestimate the absolute magnitude of the unknown “true” mean effect size?

It might seem that there is no way to answer this question. The whole point of a meta‐analysis is to summarize all available data on the effect size of interest. If the available data are insufficient to produce a reasonably precise estimate of the unknown “true” mean effect size, one might think that the only solution is to await the publication of additional data.

Here I address this question using meta‐meta‐analysis. A meta‐meta‐analysis, also called second‐order meta‐analysis, is a meta‐analysis of meta‐analyses (Barto & Rillig, 2012; Busch & Friede, 2018; Cafri et al., 2010; Costello & Fox, 2022; Da Costa et al., 2015; Eisend & Tarrahi, 2016; Eisend, 2015; Fanelli et al., 2017; Fanshawe et al., 2017; Jennions & Møller, 2002; Martin et al., 2022; Mathur & VanderWeele, 2021; Peterson, 2001; Rebar et al., 2015; Sáiz‐Vazquez et al., 2020; Steenbergen‐Hu et al., 2016; Tan et al., 2020; van Aert et al., 2019; Yang et al., 2022; Young, 2017). Meta‐meta‐analyses can be used for various purposes. Meta‐meta‐analyses can be used to estimate the prevalence and/or typical magnitude of publication biases (Barto & Rillig, 2012; Costello & Fox, 2022; Fanelli et al., 2017; Fanshawe et al., 2017; Jennions & Møller, 2002; Mathur & VanderWeele, 2021; Pietschnig et al., 2019; van Aert et al., 2019). Different meta‐analyses of the same effect can be combined into a meta‐meta‐analysis to obtain a more precise estimate of the mean effect size, estimate heterogeneity in mean effect size among meta‐analyses, and improve power to detect effects of moderator variables associated with variation in effect size (Castellanos & Verdú, 2012; Da Costa et al., 2015; Duke et al., 2017; Eisend & Tarrahi, 2016; Jüni et al., 2002; Martin et al., 2022; Mingebach et al., 2018; Peterson, 2001; Rebar et al., 2015; Sáiz‐Vazquez et al., 2020; Steenbergen‐Hu et al., 2016; Tan et al., 2020; Young, 2017). Meta‐meta‐analysis can be used to compare the statistical properties of different meta‐analyses, such as Type I error rate, heterogeneity, and statistical power (Cafri et al., 2010; Senior et al., 2016; Turner et al., 2013).

Here, I use hierarchical random effects meta‐meta‐analysis to estimate the variance in effect size among meta‐analyses, among primary research studies within meta‐analyses, and among effect sizes within primary research studies. The key outputs of such a meta‐meta‐analysis, for purposes of this paper, are best linear unbiased predictions (BLUPs) of the true mean effect size for every meta‐analysis. These BLUPs are shrinkage estimates. Hierarchical random effects meta‐meta‐analysis subtracts out within meta‐analysis sources of variation in effect size, thereby shrinking the predicted mean effect size for each meta‐analysis towards the grand mean. Meta‐meta‐analysis can be thought of as a way of adjusting the estimated mean effect size for each meta‐analysis by using information from other meta‐analyses of other topics. A meta‐analytic mean effect size that is much more positive, or much more negative, than those from other ecological meta‐analyses likely is the product of sampling error. One can think of the BLUP of the meta‐analytic mean effect size as a prediction of what the mean effect size would be for a given meta‐analysis, if many additional primary research studies were conducted.

It might seem implausible that one could improve the estimated mean effect size for any given meta‐analysis using information from meta‐analyses of other, unrelated topics. However, the statistical rationale for shrinkage estimation does not depend on whether all the data included in the analysis concern the “same” or “related” topics (see Efron & Morris, 1977 for an accessible discussion of this point). Rather, shrinkage estimation of population means will be more effective, the more homogeneous the populations are—that is, the less variance there is among the true population means (Efron & Morris, 1977). But even in the presence of among‐population heterogeneity, shrinkage should still improve the average accuracy of the estimated means (Efron & Morris, 1977). Shrinkage estimation of population means also will be more effective, the more populations are considered (Efron & Morris, 1977). The goal of shrinkage estimation is to reduce the total estimation error for a set of multiple population means. The shrinkage estimate of any particular population means will not necessarily be closer to the unknown true value than the unshrunken estimate. The degree to which shrinkage estimation of meta‐analytic mean effect sizes reduces the total error of the unshrunken means is an empirical question. Below, I demonstrate a way to empirically validate whether shrinkage estimates of meta‐analytic means improve on the unshrunken means.

Using a compilation of effect sizes from 467 ecological meta‐analyses (Costello & Fox, 2022), I conducted a meta‐meta‐analysis of 95 ecological meta‐analyses using the log‐transformed response ratio as the effect size measure, and a meta‐meta‐analysis of 118 ecological meta‐analyses using Fisher's *z*‐transformed correlation coefficient as the effect size measure. Both meta‐meta‐analyses revealed that the mean effect size from the typical (median) ecological meta‐analysis should be shrunk by ~10% in absolute magnitude. Some mean effect sizes—primarily large‐magnitude means from small meta‐analyses—should be shrunk by >50%. Simulated data and an empirical validation exercise give confidence that shrinkage estimates of mean effect size from meta‐meta‐analysis improve on unshrunken means.

## 2 METHODS

### 2.1 Data compilation

Here I summarize the key details of the data compilation methods. See Costello and Fox (2022) for further details and PRISMA diagram. Text in this subsection is lightly paraphrased from Costello and Fox (2022).

Costello and Fox (2022) conducted a systematic search for ecological meta‐analyses in 2020. For purposes of this paper, a “meta‐analysis” is a set of effect sizes that the authors of a meta‐analysis paper averaged together to obtain a mean effect size and that were not used in any other meta‐analysis from the same paper. Some meta‐analysis papers report multiple meta‐analyses. For instance, several meta‐analysis papers in community ecology report two separate meta‐analyses, one reporting effect sizes on total abundance, and the other reporting effect sizes on some measure of species diversity. Some meta‐analysis papers report multiple meta‐analyses, some of which used a subset of the effect sizes used in others. In such cases, I used only the most inclusive meta‐analysis reported. Meta‐analysis authors varied in their choices as to which data to include in their meta‐analyses. For instance, some meta‐analysis authors conducted separate meta‐analyses of different measures of fitness or performance (e.g., growth, survival, reproduction). Other meta‐analysis authors included different fitness or performance measures in the same meta‐analysis and then tested for variation in mean effect size among different fitness or performance measures. I always followed the choices of the meta‐analysis authors as to which data to include. The completed compilation included 467 meta‐analyses from 232 meta‐analysis papers. These meta‐analyses included a total of 14,634 primary studies, reporting 111,320 effect sizes. Meta‐analyses ranged in size from 4–9400 effect sizes from 3–369 studies, published over 1–84 years. The typical meta‐analysis included 64 effect sizes from 24 studies (medians; means were 239 and 40, respectively), published over a period of 21 years (median; mean was 22 years).

### 2.2 Data analysis

I conducted two hierarchical random effects meta‐meta‐analyses. Each hierarchical random effects meta‐meta‐analysis partitioned the total variance in effect size into components attributable to random effects of variation among meta‐analyses, variation among studies within meta‐analyses, variation among effect sizes within studies, and sampling error. The random effects model assumed that mean effect sizes for the meta‐analyses were sampled from a normal distribution characterized by a grand mean and a variance. The mean effect sizes for a given study within a given meta‐analysis were assumed to deviate from the mean for that meta‐analysis by a random deviation drawn from a normal distribution with a mean of zero and a variance specific to that meta‐analysis. Effect sizes within a study were assumed to deviate from the study‐specific mean by random deviates drawn from a normal distribution with a mean of zero and a study‐specific variance. The key outputs of each meta‐meta‐analysis are the estimated mean effect sizes for the meta‐analyses (best linear unbiased predictors, known as BLUPs). These BLUPs are shrinkage estimates: accounting for random variation within and among meta‐analyses shrinks the estimated true mean effect size for each meta‐analysis towards the grand mean. Large‐magnitude meta‐analytic means (that is, those farthest from the grand mean) will tend to experience more shrinkage.

I conducted two meta‐meta‐analyses: one of all the meta‐analyses in the compilation that used the log‐transformed response ratio as the effect size measure, and one of all the meta‐analyses in the compilation that used Fisher's *z*‐transformed correlation coefficient as the effect size measure. All effect size measures are unitless ratios, and so in principle meta‐analyses using different effect size measures could be included in the same meta‐meta‐analysis. I did not do this, for two reasons. First, different effect size measures are unitless ratios of variables measured in different units, and so have different statistical properties and scientific interpretations. Second, it would have been computationally infeasible to include all 467 meta‐analyses in the same meta‐meta‐analysis, at least with the computational resources available to me. Indeed, it proved computationally infeasible for me to conduct a meta‐meta‐analysis just of meta‐analyses using Hedges' *d* or *g* as the effect size measure.

To quantify and summarize the amount of shrinkage produced by each meta‐meta‐analysis, I conducted separate hierarchical random effects meta‐analyses, each of which estimated variation in effect size attributable to variation among studies, among effect sizes within studies, and sampling error. Each of these separate meta‐analyses provided an unshrunken mean effect size, which can be compared with the corresponding shrunken mean (BLUP) from the appropriate meta‐meta‐analysis.

### 2.3 Validating meta‐meta‐analysis

I used two approaches to validate whether BLUPs from meta‐meta‐analysis improve on unshrunken mean effect sizes. The first approach used simulated data. I generated simulated data from 50 meta‐analyses, comprised of 5, 10, 20, 30, or 50 primary research studies (10 meta‐analyses of each size). Each primary research study reported two effect sizes. The effect sizes were Fisher's *z*‐transformed correlation coefficients, each based on a sample of 20 observations. The true mean effect size for each meta‐analysis was sampled randomly from a normal distribution with a mean of 0.1 and a standard deviation of 0.1. The true mean effect size for each study within a given meta‐analysis equaled the meta‐analytic mean, plus a random deviation sampled from a normal distribution with a mean of 0 and a standard deviation of 0.3. Each effect size equaled the mean of the study from which it came, plus a random deviation sampled from a normal distribution with a mean of 0 and a standard deviation of 0.3. The sampling variance for each effect size equaled 1/(20–3). I fit 50 separate hierarchical random effects meta‐analyses, each of which estimated the mean effect size, as well as partitioning the variance in effect size into different sources (variance among studies, variance among effect sizes within studies, sampling variance). I also fit a hierarchical random effects meta‐meta‐analysis that estimated a grand mean effect size, and partitioned variance in effect size into variance among meta‐analyses, variance among studies within meta‐analyses, variance among effect sizes within meta‐analyses, and sampling variance. The BLUPs from this meta‐meta‐analysis are shrunken estimates of mean effect size for each meta‐analysis, that subtract out other sources of variation in effect size besides variation in the true mean effect size among meta‐analyses.

The simulation results show that meta‐meta‐analysis can be helpful, but on their own, they do not establish that meta‐meta‐analysis is always helpful. One could change the simulation parameters to produce scenarios in which shrinkage improved the average estimation error very little, and scenarios in which it improved the average estimation error even more. To establish whether meta‐meta‐analysis is likely to be helpful when applied to ecological meta‐analyses, I conducted an empirical validation exercise.

The goal of the validation exercise is to check whether BLUPs of mean effect size for small ecological meta‐analyses accurately predict mean effect sizes after many additional studies have been conducted. The validation exercise used 12 of the largest meta‐analyses in my compilation, ranging in size from 114–243 primary research studies. These were all the meta‐analyses in my compilation that used the log‐transformed response ratio as the effect size measure, and that comprised >100 but <300 primary research studies. Using meta‐analyses with >300 primary research studies proved computationally infeasible on the computer available to me. I first conducted a hierarchical random effects meta‐meta‐analysis of all 12 meta‐analyses, using the same model structure and statistical assumptions as for the meta‐meta‐analyses described in the previous subsection. This meta‐meta‐analysis provided BLUPs of the “true” mean effect sizes. Note that these BLUPs are very close to the unshrunk meta‐analytic means, as expected because these 12 meta‐analyses all are very large. Then, I repeated the meta‐meta‐analysis 12 times, each time omitting all but the first 10 published studies from one of the 12 meta‐analyses. This procedure converted one of the 12 large meta‐analyses into an artificial small meta‐analysis. The BLUPs for these small meta‐analyses are shrinkage estimates of the true meta‐analytic mean effect sizes, informed by data from the other, larger meta‐analyses included in the meta‐meta‐analysis. Finally, I also conducted 12 separate meta‐analyses, each using just the effect sizes from the first 10 published studies comprising one of the 12 large meta‐analyses. These 12 separate meta‐analyses provided unshrunken estimates of the true mean effect sizes. I compared the shrunken and unshrunken estimates of the true mean effect sizes to the mean effect sizes from the meta‐meta‐analysis of all 12 large meta‐analyses.

I fit all meta‐analyses and meta‐meta‐analyses reported in this paper via restricted maximum likelihood (REML), assuming a compound symmetric variance–covariance matrix. Meta‐meta‐analyses additionally assumed a sparse variance–covariance matrix. I conducted all statistical analyses using R 3.6.3 running within R Studio 1.3.1093 (R Core Team, 2020). I fit the meta‐analyses and meta‐meta‐analyses using the rma.mv function from the metafor package, version 2.4‐0 (Viechtbauer, 2010).

## 3 RESULTS

**FIGURE 1:**
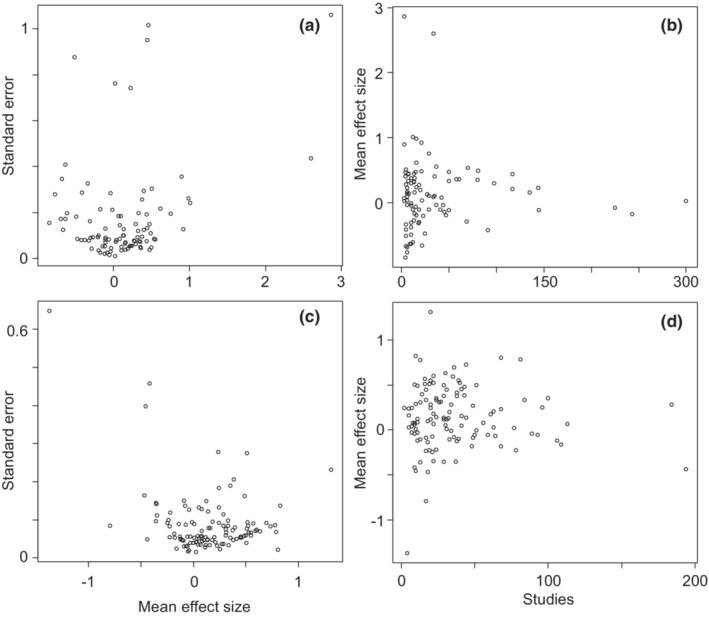
(a, c) Mean effect size vs. standard error of the mean, for meta‐analyses using either the log‐transformed response ratio as the effect size measure (a) or Fisher's *z*‐transformed correlation coefficient (c). (b, d) Mean effect size vs. the number of studies in the meta‐analysis, for meta‐analyses using either the log‐transformed response ratio as the effect size measure (b) or Fisher's *z*‐transformed correlation coefficient (d).

Figure 1 illustrates why we might expect shrinkage estimation to improve estimates of mean effect size in ecological meta‐analyses. Plotting mean effect sizes from ecological meta‐analyses against their standard errors yields a funnel shape: precisely estimated mean effect sizes all fall close to zero, whereas imprecisely estimated means are scattered much more widely around zero (Figure 1a,c). Further, the most imprecisely estimated mean effect sizes, some of which are very large in absolute magnitude, come from the smallest meta‐analyses (Figure 1b,d). Figure 1 strongly suggests that the largest magnitude mean effect sizes reported in ecological meta‐analyses are at least partially the product of sampling error. These meta‐analytic means could be made more accurate in the aggregate if they were shrunk to some degree.

**FIGURE 2:**
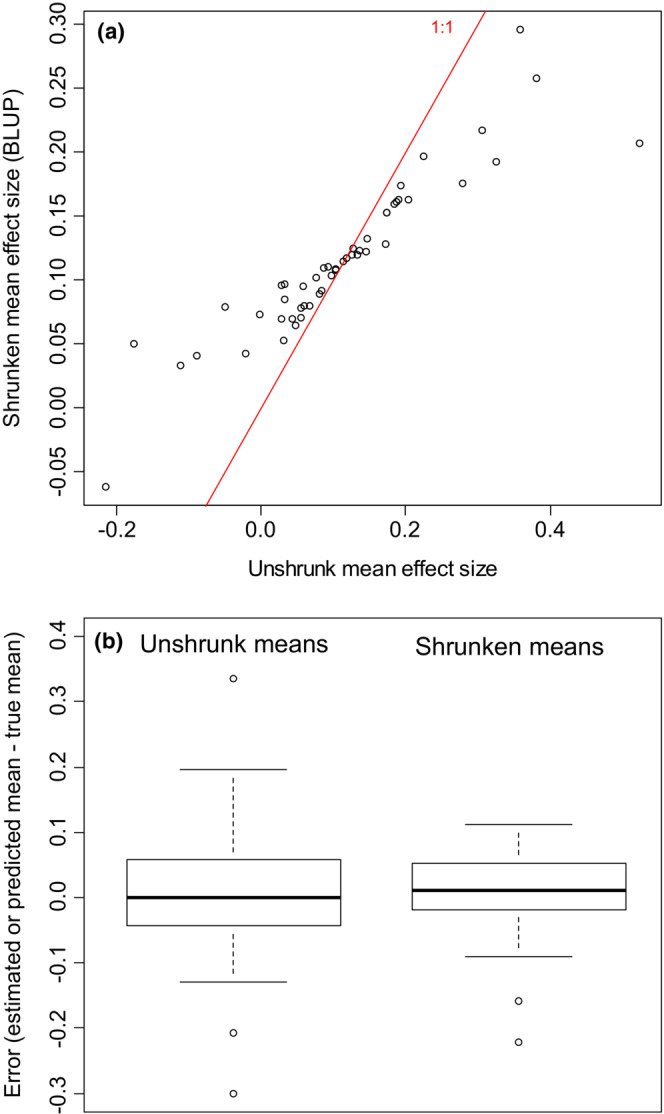
Results of an illustrative simulated meta‐meta‐analysis. (a) Estimated mean effect size from each of 50 simulated meta‐analyses (*x*‐axis), vs. best linear unbiased predictions (BLUPs) of mean effect size from a hierarchical random effects meta‐meta‐analysis (*y*‐axis). Solid red line is the 1:1 line. The meta‐meta‐analysis shrinks larger‐magnitude means further towards the grand mean, reducing very positive means and increasing very negative means. (b) Boxplots of the errors (estimated or predicted mean minus true mean) for meta‐analyses and for BLUPs from the meta‐meta‐analysis. The errors of the BLUPs are smaller on average.

Figure 2a illustrates the shrinkage produced by the simulated meta‐meta‐analysis. Meta‐meta‐analysis shrinks large‐magnitude meta‐analytic means towards the grand mean so that there is less variance among the BLUPs than there is among the meta‐analytic means (Figure 2a). Figure 2b shows that this shrinkage improves the estimated means, in aggregate. On average, the shrunken means (BLUPs) are closer to their true values than are the unshrunken meta‐analytic means. Shrinkage does not necessarily move any particular meta‐analytic mean closer to its true value, but shrinkage reduces average estimation error for the entire set of 50 means.

**FIGURE 3:**
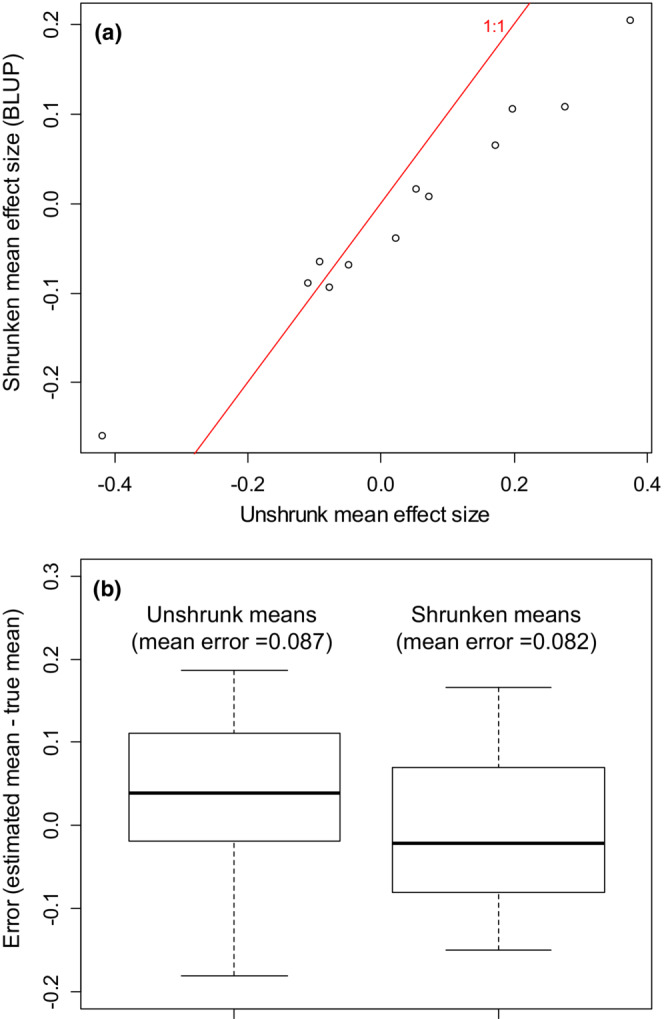
Results of the empirical validation exercise. (a) Estimated mean effect sizes for 12 artificial small meta‐analyses (*x*‐axis), vs. best linear unbiased predictions (BLUPs) of mean effect size from a hierarchical random effects meta‐meta‐analysis (*y*‐axis). Solid red line is the 1:1 line. The meta‐meta‐analysis shrinks larger‐magnitude means further towards the grand mean. (b) Boxplots of the errors (estimated or predicted mean minus “true” mean) for meta‐analyses and for BLUPs from the meta‐meta‐analysis. The errors of the BLUPs are slightly smaller on average.

Figure 3a illustrates the shrinkage produced in the empirical validation exercise. Meta‐meta‐analysis shrinks the estimated mean effect sizes from the artificial small meta‐analyses (Figure 3a). Figure 3b shows that this shrinkage slightly improves the estimated means, in aggregate. On average, the shrunken means (BLUPs) for the artificial small meta‐analyses are closer to their “true” values (i.e., values with all studies included) than are the unshrunken means for the artificial small meta‐analyses (Figure 3b). As in the simulated example, shrinkage does not necessarily move any particular meta‐analytic mean closer to its true value, but shrinkage does slightly reduce the average estimation error for the entire set of 12 means. Note that the empirical validation exercise is conservative, in that it considers a scenario unfavorable to effective shrinkage estimation. The empirical validation exercise only considers 12 means; the effectiveness of shrinkage estimation should increase with the number of population means considered, all else being equal (Efron & Morris, 1977). The empirical validation exercise also does not include any small meta‐analyses reporting extremely large or extremely small mean effect sizes. The unshrunken meta‐analytic means for the artificial small meta‐analyses range from −0.42 to +0.38 (Figure 3a). Small ecological meta‐analyses report mean effect sizes spanning a much wider range (Figure 1a,b).

**FIGURE 4:**
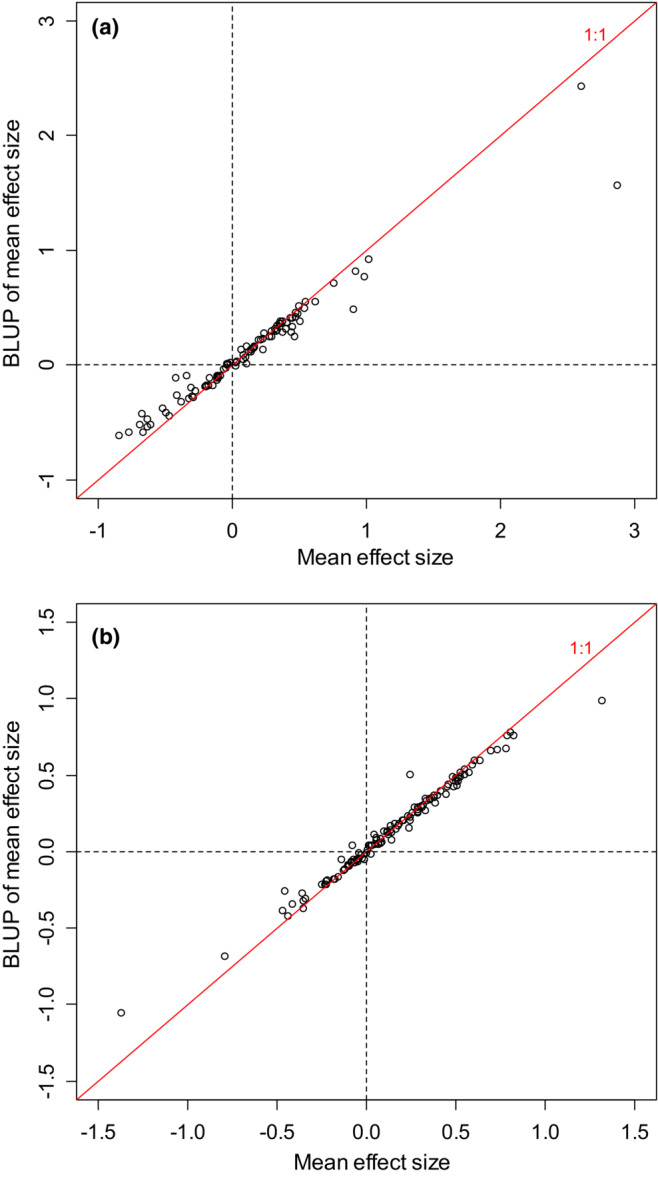
Best linear unbiased predictor (BLUP) of mean effect size, vs. mean effect size. Each point gives data for one meta‐analysis. In each panel, dashed lines mark means of zero, and the red solid line is the 1:1 line. (a) Meta‐analyses using the log‐transformed response ratio as the effect size measure. (b) Meta‐analyses using Fisher's *z*‐transformed correlation coefficient as the effect size measure.

Figure 4 plots BLUPs of mean effect size from the meta‐meta‐analyses vs. the corresponding unshrunken meta‐analytic means. As one would expect, small magnitude meta‐analytic means experience little shrinkage, whereas large magnitude meta‐analytic means experience substantial shrinkage. We can express the amount of shrinkage as a percentage of the magnitude of the unshrunken mean: 100 × abs(Y − X)/abs(X), where Y is the BLUP, X is the unshrunken meta‐analytic mean effect size, and abs() denotes the absolute value operator. Meta‐meta‐analysis shrinks the median meta‐analytic mean by 11% (for meta‐analyses using the log‐transformed response ratio), or 10% (for meta‐analyses using Fisher's *z*‐transformed correlation coefficient). The distribution of shrinkage is skewed; the majority of meta‐analytic means shrink very little, but a minority shrinks substantially (Figure 4). Twenty‐four out of 213 meta‐analytic means shrink by >50% (Figure 4).

## 4 DISCUSSION

The premise of meta‐analysis is that any individual effect size is likely to be noisy and unrepresentative. For that reason, a single effect size on its own provides very little information about the typical effect size. Only by combining many noisy effect sizes from many primary research studies into a meta‐analysis can we obtain a precise, accurate estimate of the true mean effect size. The same logic applies to meta‐analyses. A single meta‐analysis on its own does not provide complete, accurate information about the true mean effect size, particularly if the meta‐analysis is small. By combining information from many meta‐analyses into a meta‐meta‐analysis, we can obtain more accurate estimates of mean effect sizes. Previous research used shrinkage estimation to improve effect estimates within single studies in ecology (e.g., Badri et al., 2020). Here I use the same broad approach to improve estimates of meta‐analytic mean effect sizes.

Shrinkage estimation works despite heterogeneity among meta‐analyses. Different meta‐analyses concern different topics, which are studied using different methods. The true mean effect size therefore varies substantially among meta‐analyses. BLUPs for meta‐analyses using the log‐transformed response ratio as the effect size measure ranged from −1.17‐2.52, implying that the mean response ratios ranged from exp(−1.17) = 0.31 up to exp(2.52) = 12.43. BLUPs for meta‐analyses using Fisher's *z*‐transformed correlation as the effect size measure ranged from −1.55‐1.05, implying that the mean correlation coefficients ranged from −0.91 up to 0.78. But different ecological meta‐analyses nevertheless still provide useful context for one another. Analogously, ecological meta‐analyses typically exhibit substantial heterogeneity in effect size within and among primary research studies (Senior et al., 2016). The mere fact that the true mean effect size varies within and among primary research studies does not mean that those studies should not be included in the same meta‐analysis. By the same token, the fact that the true mean effect size varies among ecological meta‐analyses does not mean that they should not be included in the same meta‐meta‐analysis.

However, although heterogeneity among meta‐analyses does not invalidate their inclusion in the same meta‐meta‐analysis, it does limit the amount of shrinkage that the meta‐meta‐analysis will produce. Meta‐meta‐analysis shrunk the mean effect size for the typical (median) ecological meta‐analysis by just 10%—a fairly modest amount of shrinkage for most purposes. One reason for that modest shrinkage is the substantial heterogeneity in mean effect size among meta‐analyses. Because of this substantial heterogeneity among meta‐analyses in their true mean effect sizes, one cannot greatly improve the average estimation error by shrinking all of the means towards the grand mean (or any other single value). The optimal amount of shrinkage therefore will be fairly small (Efron & Morris, 1977; Stein, 1981).

There are two other reasons why meta‐meta‐analysis produced only modest shrinkage of mean effect size for the majority of ecological meta‐analyses. First, many ecological meta‐analyses, including many small ones, report small magnitude mean effect sizes (i.e., close to zero). Meta‐meta‐analysis will not shrink small means very much. Second, hierarchical random effects meta‐meta‐analysis assumes that future studies on any given topic will sample effect sizes from the same (possibly heterogeneous) distribution as previous studies. This assumption usually holds but not always. Approximately 3%–5% of ecological meta‐analyses exhibit true directional trends in mean effect size over time as more and more studies are published (Costello & Fox, 2022). Those directional trends arose because primary research studies were published in nonrandom order with respect to the effect sizes they reported, usually with larger‐magnitude effect sizes being published earlier (Costello & Fox, 2022). Thus, for a small fraction of ecological meta‐analyses, a hierarchical random effects meta‐meta‐analysis will not shrink the mean effect size enough, because the true mean effect size will itself shrink over time.

Shrinkage estimation is a way of improving the average accuracy of a set of estimates but does not necessarily improve the accuracy of every estimate (Efron & Morris, 1977, Stein, 1981). Meta‐meta‐analysis is not necessarily a way to identify which specific meta‐analyses provide inaccurate overestimates of the magnitude of the mean effect size. However, it could be used to identify candidate cases of inaccuracy and the required additional data and further analyses to verify. The most obvious candidates to have overestimated the magnitude of the mean effect size are small meta‐analyses that reported large magnitude means.

These results build on those of Yang et al. (2022). Yang et al. (2022) showed that meta‐analyses of global change typically have moderate power, implying that the absolute magnitudes of the mean effect sizes they report are somewhat overestimated on average. Here I quantify that overestimation, for meta‐analyses covering a wider range of ecological topics.

One possible application of these results is to shrink estimated mean effect sizes from future ecological meta‐analyses, particularly those for which interest centers on the precise magnitude of the mean effect size, as opposed to merely testing the null hypothesis that the true mean effect size is zero. There are arguments for shifting the focus of much ecological research away from null hypothesis testing and towards precise, accurate estimation of statistical parameters (Anderson et al., 2000; Fidler et al., 2006; Halsey, 2019; Hobbs & Hilborn, 2006; Johnson, 1999; Lemoine et al., 2016; Nakagawa & Cuthill, 2007). Precisely and accurately estimating statistical parameters requires making full use of all relevant information. Meta‐meta‐analysis shows how relevant information can come not just from other studies of the same topic but from studies of other topics as well.

## AUTHOR CONTRIBUTIONS

**Jeremy W. Fox:** Conceptualization (lead); data curation (lead); formal analysis (lead); funding acquisition (lead); investigation (lead); methodology (lead); project administration (lead); resources (lead); software (lead); supervision (lead); validation (lead); visualization (lead); writing – original draft (lead); writing – review and editing (lead).

## Data Availability

The full dataset is available on the Dryad repository, DOI: 10.5061/dryad.zkh1893b7.
